# Intra-breath changes in respiratory mechanics are sensitive to history of respiratory illness in preschool children: the SEPAGES cohort

**DOI:** 10.1186/s12931-024-02701-9

**Published:** 2024-02-24

**Authors:** Valérie Siroux, Anne Boudier, Sarah Lyon-Caen, Joane Quentin, Yoann Gioria, Zoltán Hantos, Rémy Slama, Isabelle Pin, Sam Bayat

**Affiliations:** 1 University Grenoble Alpes, Inserm U1209, CNRS UMR5309, Team of Environmental Epidemiology Applied to the Development and Respiratory Health, Institute for Advanced Biosciences, IAB, Grenoble, 38000 France; 2 CHU Grenoble-Alpes, Grenoble, France; 3 Department of Anesthesiology and Intensive Therapy, Semmelweis University, Budapest, Hungary; 4 Department of Technical Informatics and Engineering, University of Szeged, Szeged, Hungary; 5 University Grenoble Alpes, Dept. of Pulmonology, STROBE Inserm UA7 Laboratory, Grenoble, France

**Keywords:** Epidemiology, Forced oscillation technique, Respiratory resistance, Respiratory reactance, Asthma, Preschool children

## Abstract

**Background:**

Intra-breath oscillometry has been proposed as a sensitive means of detecting airway obstruction in young children. We aimed to assess the impact of early life wheezing and lower respiratory tract illness on lung function, using both standard and intra-breath oscillometry in 3 year old children.

**Methods:**

History of doctor-diagnosed asthma, wheezing, bronchiolitis and bronchitis and hospitalisation for respiratory problems were assessed by questionnaires in 384 population-based children. Association of respiratory history with standard and intra-breath oscillometry parameters, including resistance at 7 Hz (R_7_), frequency-dependence of resistance (R_7 − 19_), reactance at 7 Hz (X_7_), area of the reactance curve (AX), end-inspiratory and end-expiratory R (R_eI_, R_eE_) and X (X_eI_, X_eE_), and volume-dependence of resistance (ΔR = R_eE_-R_eI_) was estimated by linear regression adjusted on confounders.

**Results:**

Among the 320 children who accepted the oscillometry test, 281 (88%) performed 3 technically acceptable and reproducible standard oscillometry measurements and 251 children also performed one intra-breath oscillometry measurement. Asthma was associated with higher R_eI_, R_eE_, ΔR and R_7_ and wheezing was associated with higher ΔR. Bronchiolitis was associated with higher R_7_ and AX and lower X_eI_ and bronchitis with higher R_eI_. No statistically significant association was observed for hospitalisation.

**Conclusions:**

Our findings confirm the good success rate of oscillometry in 3-year-old children and indicate an association between a history of early-life wheezing and lower respiratory tract illness and lower lung function as assessed by both standard and intra-breath oscillometry. Our study supports the relevance of using intra-breath oscillometry parameters as sensitive outcome measures in preschool children in epidemiological cohorts.

**Supplementary Information:**

The online version contains supplementary material available at 10.1186/s12931-024-02701-9.

## Introduction

Early lung development is an important determinant of long-term lung function, and of general health [1–4]. A better understanding the determinants of early-life lung function, when the lung is particularly vulnerable to damage during this critical period of lung development, is needed. However, few birth cohorts have objectively measured respiratory function in preschool children, mainly because reliable and reproducible spirometry measures are difficult to obtain in children younger than 5 years due to their difficulty to perform forced spirometry.

Oscillometry, referred to as forced oscillation technique (FOT) previously, has been successfully used in healthy preschool children and unsedated infants, to assess lung function and the effect of prenatal environmental exposures [5–8]. Oscillometry requires no active participation or breathing maneuvers, and its standard or spectral version allows computing the average respiratory impedance (Zrs) in terms of its main components: resistance (R) and reactance (X) at multiple oscillation frequencies, during a period of quiet tidal breathing. A number of studies, including both clinical and population-based studies, have demonstrated the utility of oscillometry for assessing lung function in children [8–15]. However, spectral oscillometry does not reveal the variations in respiratory mechanics that occur within the breathing cycle. In patients with chronic obstructive pulmonary disease, it has been shown that intra-breath respiratory system reactance provides an accurate and reliable measure to detect expiratory flow limitation [16]. Recent studies have demonstrated that intra-breath variations in R and X are more sensitive than spectral oscillometry in detecting airway obstruction and mechanical heterogeneity both in children [17], and adults [18, 19]. Moreover, intra-breath oscillometry has been shown to predict the risk of lower respiratory tract illness (LRTI) in healthy new-born African infants [20]. However, data on the impact of a history of LRTI, wheezing or asthma in the first years of life, on later lung function in population-based preschool children is lacking [21]. A recent study in the Drakenstein Child Health Study showed higher airway intra-breath resistance measures in 5-year-old children with early and recurrent wheeze as compared with children who had never wheezed [22]. 

We aimed to assess the associations between a history of asthma, wheezing or LRTI and both standard and intra-breath measures of respiratory mechanics, using oscillometry. We made the hypothesis that intra-breath changes in R and X are more sensitive than standard oscillometry measures in detecting a history of respiratory illness in young children.

## Methods

### Population

This study relies on the *Suivi de l’Exposition à la Pollution Atmosphérique durant la Grossesse et Effets sur la Santé* (SEPAGES) couple-child cohort. The study design, visit structure and assessments have been described in detail previously [23]. Women aged 18 years or older were recruited before the 19th gestational week of pregnancy, from 2014 to 2017 in the Grenoble area, France. Women were followed up during pregnancy and children were followed up at two months, one year, two years and three years. The present study relies on 384 children who participated in the 3-year visit.

### Lung function assessed by oscillometry at the 3-year visit

Oscillometry was measured with a commercial device (tremoFlo C-100, Thorasys Systems Montreal, QC, Canada) complying with current European Respiratory Society standards [24]. The device, calibrated daily using a test resistance, superimposes a pseudo-random oscillatory signal containing frequencies from 7 to 41 Hz on a tidal breathing. This oscillatory signal was chosen in order to avoid the corrupting effect of breathing frequency which is higher in 3-year-olds than in adults, in whom the 5–37 Hz signal is used. The measurements were performed in seated position with the head slightly extended, a nose clip to avoid air leakage, lips firmly closed around the mouthpiece and cheeks and chin maintained by the technician to minimise the effects of shunting the oscillations by the mouth walls. Measurements were excluded in case of leakage, swallowing, glottis closure, vocalization or obstruction of the mouthpiece by the tongue. After a period of about 30 s during which the child got used to the device, three to five acceptable 16-s measurements, with one minute resting intervals in between, were obtained and averaged. The measurements were performed at least 15 days away from any respiratory infection. The parameters recorded and analyzed were reactance at 7 Hz (X_7_), resistance at 7 Hz (R_7_), frequency dependence of the resistance between 7 and 41 Hz (R_7 − 19_), a parameter that reflects the heterogeneous obstruction of the distal bronchi, and the area of the reactance curve (AX). Reproducibility of oscillometry measures was assessed by a coefficient of variation (CV) for R_7_ ≤ 15% between the 3 technically acceptable measurements [24]. For each oscillometry parameter, the individual mean value was calculated on the acceptable and reproducible maneuvers. For X_7_ and R_7_, Z-scores were computed based on the reference equations estimated from the children’s height for descriptive purposes, to allow a direct external comparison of the X_7_ and R_7_ distribution [25].

Additionally, by using the research modality of the tremoFlo, a 10 Hz tracking signal was applied in a 20 s measurement to assess the intra-breath changes in Zrs. The recordings of pressure (P), flow (V’) and volume (V) were imported in a custom-made software. Fast Fourier transformation was used to compute R and X at 10 Hz (R_10_ and X_10_, respectively), after band-pass filtering of V’ and P between 8 and 12 Hz. Linear interpolation was used to establish zero-crossings of V’, i.e. the time of end expiration (eE) and end inspiration (eI), and the corresponding R10 and X10 values. Since the latter represent zero-flow values, they approximate resistance and reactance of the respiratory system without the influence of flow nonlinearities in the upper airways. This allowed to calculate the corresponding end-expiratory and end-inspiratory R (ReI, ReE) and X (XeI, XeE) and the change in R and X between eE and eI (ΔR and ΔX, respectively). Supplementary Figure S1 shows examples of the intra-breath data in a child with normal airway resistance and in a child with elevated airway resistance.

### Respiratory diseases and allergy-related outcomes

Parents of the children replied to a respiratory questionnaire administered by an interviewer at 2 months, 1 year, 2 years and 3 years, which is based on the ISAAC questionnaire, classifying into groups of *ever wheezing* (no/yes), *ever bronchitis* (no/yes), *ever bronchiolitis* (no/yes) *and ever asthma* (no/yes). *Ever wheezing and bronchiti*s over the first 3 years of life were defined by a positive response to the questions: “In the last 12 months (or since birth for the 2- month questionnaire), has your child had wheezing in the chest?” and “In the last 12 months (or since birth for the 2-month questionnaire), has your child had bronchitis?” in at least one of the questionnaires. Doctor diagnosed asthma was defined by a positive answer to: “In the last 12 months (or since birth for the 2-month questionnaire), has your child had asthma” and to: “Was the asthma diagnosis confirmed by a medical doctor?” in at least one of the questionnaires. Bronchiolitis over the first two years of life was defined by a positive response to: “In the last 12 months (or since birth for the 2-month questionnaire), has your child had a bronchiolitis” in at least one of the 2-month, 1-year and 2-year questionnaires.

In addition to the respiratory questionnaires administered by an interviewer at 2 months, 1 year, 2 years and 3 years, the parents of the children were invited to reply to a self-completed questionnaire at 3, 6, 9, 15, 18 and 21 months including information on hospitalisation. Hospitalisation for respiratory problem over the first 3 years of life were defined by a positive response to the question: “Has your child been hospitalised (including day hospitalisation)” and either wheeze, asthma attack, coughing episode and respiratory infection as reason for hospitalisation in at least one of these questionnaires.

Allergic sensitization was defined by at least 1 positive skin prick test (mean wheal diameter of 3 mm or more) among the 5 allergen sources tested (cat, *D pteronyssinus*, *D farinae*, birch, Timothy grass / grass) at the 3-year visit.

### Statistical analysis

The unadjusted association between each oscillometry parameter and individual characteristics (age, sex, height, weight, parental educational level, gestational duration, breastfeeding, tobacco smoke exposure (child exposure to passive smoking in the first 3 years of life, assessed by questionnaire), parental history of asthma and child allergic sensitization) were first evaluated using t-test for binary variables and one-way ANOVA for continuous individual characteristic coded in three groups according to terciles (allowing us to address the linearity of the association). Then, univariate linear regression models were conducted for individual characteristics retained in the adjusted models.

The association between each oscillometry parameter measured at three years of age (considered as the dependent variable, “Y”) and each history of respiratory disease over the first three years of life (considered as the independent variable, “X”) was analysed using separate linear regression models. The M0 models were adjusted on age (continuous), sex and height (continuous). The main models were further adjusted on potential confounders identified from the literature and by using a directed acyclic graph (Supplementary Figure S2): tobacco smoke exposure, i.e. child passive smoking (defined by: ever since birth vs. never; maternal active smoking (at least one cigarette per day) or presence of relatives who smoke inside the child’s home, in the 12-month or 36-month maternal questionnaire), birthweight (continuous), breastfeeding (never or less than 2 months vs. ≥2 months), parental asthma (binary) and child allergic sensitization (≥ 1 positive skin prick test). Each oscillometry parameter was re-scaled (divided by its standard deviation) to allow comparisons of the effect estimates across the different oscillometry parameters. For each linear regression, analysis of the residual plots assessing the linearity of the data, as well as the homogeneity of residual variance and the normality of the residuals is presented in supplementary Figure S3.

Missing data on confounders (rate of missing data was < 1% for all confounders except for parental history of asthma and child allergic sensitization for which the missing data rate was 8.3%) were imputed using a multiple imputation method (twenty simulations were performed using the fully conditional specification method (FCS)) [26]. Analyses were performed using SAS version 9.4 (SAS Institute, Cary, NC, USA). The level of significance used was 0.05. Although the main analysis consisted in 40 tests (4 “exposures” by 10 oscillometry parameters), we did not apply any formal correction for multiple comparisons because the tested associations (1) were based on strong hypotheses supported by previous data in the literature, (2) were not totally independent because of the correlation between the oscillometry parameters and between the respiratory health outcomes (Table S1 and S2, respectively). Moreover, in addition to the arbitrary level of significance, results were interpreted by looking at the consistency of associations across the different respiratory health outcomes.

## Results

### Population description

**Fig. 1:**
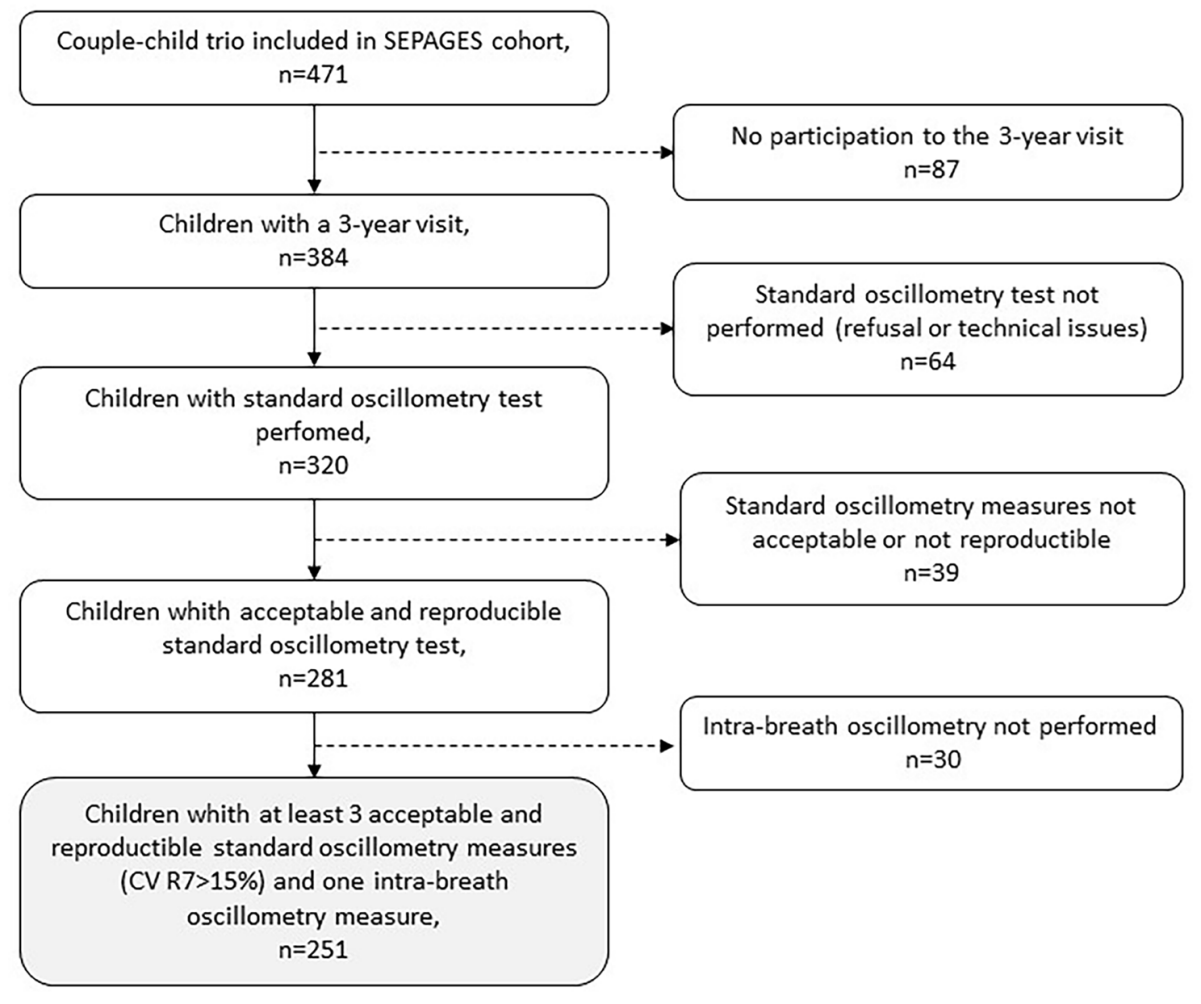
Study flowchart

Among the 384 children invited to perform oscillometry, 320 (acceptability rate = 83%) accepted and amongst them 281 (success rate = 88%) had at least 3 standard measures fulfilling the acceptability and reproducibility criteria (CV for R_7_ ≤ 15%). For intra-breath oscillometry, the acceptance criterion was ≥ 3 regular and artefact-free breathing cycles; this was fulfilled in 291 of the 320 children (mean of breathing cycles: 9.1), success rate: 91%. Data from 251 children who performed both at least one 10 Hz intra-breath and 3 standard measurements were retained in the analysis (Fig. 1). On average the included children (*n* = 251) compared to the non-included children (220 children enrolled in the SEPAGES cohort, but without oscillometry measures) had higher weight and height at birth, had more often higher parental educational levels and less often a gestational duration less than 37 weeks (Table 1). The mean (sd) age of the study population was 37.4 (3.3) months (Table 2). Ever doctor-diagnosed asthma, wheezing, bronchitis and bronchiolitis were reported in 12.0%, 33.6%, 21.1% and 37.8% of the children, respectively. In the first 3 years of life, 9.6% of the children (*n* = 24) had been hospitalised for a respiratory problem. Oscillometry indices are described in Table 3.

**Table 1:** Comparison of the included and non-included children

	Included children (n = 251)	Non-included children (n = 220)	*P* Value
	Statistic	Statistic	
Maternal age (yrs), mean ± sd	32.7 ± 3.8	32.3 ± 3.9	0.19
Parental education level, % graduate	185 (73.7)	145 (65.9)	0.06
Maternal smoking during pregnancy, %	11 (4.8)	9 (4.6)	0.91
Tobacco smoke exposure*, %	56 (22.5)	38 (20.5)	0.65
Maternal parity, % ≥1 children	145 (57.8)	112 (50.9)	0.14
Weight at birth (g), mean ± sd	3352.5 ± 413	3213.0 ± 487	< 0.01
Height at birth (cm), mean ± sd	50.3 ± 2.1	49.9 ± 2.5	0.03
Sex, % male	136 (54.2)	115 (52.5)	0.72
Gestation duration ≤ 37 weeks, %	20 (8.0)	30 (13.7)	0.04

figures presented are n(%) or mean ± sd

* Child exposure to tobacco smoke at 12 months or 36 months, as assessed from the follow-up questionnaire

**Table 2:** Description of the study population at 3 years (*n* = 251)

	**Statistic**
**Individual characteristics**	
Sex, male	115 (54.2)
Age (months)	37.4 ± 3.3
Height (cm)	95.5 ± 3.6
Weight (kg)	14.7 ± 1.6
Exposure to tobacco smoke	56 (22.5)
Parental education level, graduate level	185 (73.7)
Breastfed at 2 months	221 (88.4)
Birth weight, g	3352.5 ± 413
Gestation ≤ 37 wks	20 (8.0)
Parental history of asthma	64 (27.8)
**Child history of respiratory diseases**	
Doctor diagnosed asthma ever	30 (12.0)
Wheezing ever	84 (33.6)
Bronchiolitis*	91 (37.8)
Bronchitis ever	53 (21.1)
Hospitalisation for respiratory problem	24 (9.6)
**Allergic sensitization at the 3-year visit**	
≥ 1 positive skin prick test	27 (11.7)

figures presented are n(%) or mean ± sd

* assessed from 2 months, 1-year and 2-years questionnaires

**Table 3:** Description of the oscillometry parameters

	mean ± sd
**Standard oscillometry parameters, 3-year visit**	
R_7_ (cmH_2_O·s/L)	11.8 ± 2.2
R_7_Zscore_	0.54 ± 0.90
X_7_ (cmH_2_O·s/L)	-4.1 ± 1.2
X_7_Zscore_	0.56 ± 0.68
AX (cmH_2_O/L)	73.3 ± 33.9
R_7 − 19_ (cmH_2_O·s/L)	1.2 ± 1.1
**Intra-breath oscillometry parameters**	
R_eE_ (cmH_2_O·s/L)	13.4 ± 3.6
R_eI_ (cmH_2_O·s/L)	10.5 ± 2.6
ΔR (cmH_2_O·s/L)	2.9 ± 1.8
X_eE_ (cmH_2_O·s/L)	-3.5 ± 2.4
X_eI_ (cmH_2_O·s/L)	-3.0 ± 1.3
ΔX (cmH_2_O·s/L)	-0.49 ± 1.70

### Associations between individual child characteristics and standard oscillometry parameters at the 3-year visit

Regarding the standard oscillometry parameters, a statistically significant difference for sex was observed for R_7 − 19_, with a lower value for girls than for boys (Table 4 and Supplementary Table S3). Linear trends were observed with height and weight coded in terciles, with lower R_7_ and higher X_7_ (less negative) for taller and heavier children. As expected, the effect of height and weight were no longer significantly associated with R_7_ and X_7_ expressed in Zscore, although a trend for an association (*p* = 0.09) remained between height and R_7 − Zscore_. AX also decreased with increasing height. Higher birth weight was associated with lower R_7_. Children exposed to tobacco smoke had lower X_7_ and higher AX values. The allergic sensitization status of the children was associated with higher R_7_ and AX values. Children still breastfed at 2 months had unexpectedly lower X7 values, but no differences were observed for R7 and AX. No statistically significant differences were observed for age, parental educational level, gestational duration and parental history of asthma.

**Table 4:** Univariate associations between individual child characteristics and oscillometry parameters measured at the 3-year visit

	Age (month)		Sex (boys vs. girls)		Height (cm)		Birth weight (kg)		Tabacco smoke exposure (Y vs. N)		Breastfeeding at 2 months (Y vs. N)		Parental history of asthma (Y vs. N)		Child allergic sensitization (Y vs. N)	
	Beta ^a^ (sd)	*P* value	Beta ^a^ (sd)	*P* value	Beta ^a^ (sd)	*P* value	Beta ^a^ (sd)	*P* value	Beta ^a^ (sd)	*P* value	Beta ^a^ (sd)	*P* value	Beta ^a^ (sd)	*P* value	Beta ^a^ (sd)	*P* value
**R**_**7**_ (cmH_2_O·s/L)	0.019 (0.04)	0.65	-0.10 (0.28)	0.72	-0.19 (0.04)	**< 0.01**	-1.26 (0.32)	**< 0.01**	0.41 (0.33)	0.21	0.67 (0.43)	0.12	0.40 (0.32)	0.22	0.83 (0.44)	0.06
**X**_**7**_ (cmH_2_O·s/L)	-0.03 (0.02)	0.16	-0.14 (0.15)	0.32	0.08 (0.02)	**< 0.01**	0.24 (0.18)	0.18	-0.36 (0.17)	**0.04**	-0.52 (0.22)	**0.02**	-0.03 (0.18)	0.86	-0.21 (0.24)	0.37
**AX** (cmH_2_O/L)	0.76 (0.65)	0.25	3.92 (4.30)	0.36	-1.95 (0.58)	**< 0.01**	-12.13 (5.14)	**0.02**	12.18 (5.11)	**0.02**	9.98 (6.61)	0.13	6.56 (5.06)	0.20	14.04 (7.01)	0.05
**R**_**7 − 19**_ (cmH_2_O·s/L)	0.01 (0.02)	0.54	0.34 (0.11)	**< 0.01**	-0.01 (0.02)	0.47	0.03 (0.14)	0.84	0.19 (0.14)	0.18	0.30 (0.18)	0.10	0.13 (0.14)	0.35	0.19 (0.18)	0.30
**R**_**eE**_ (cmH_2_O·s/L)	-0.04 (0.07)	0.57	0.02 (0.45)	0.96	-0.32 (0.06)	**< 0.01**	-1.87 (0.54)	**< 0.01**	0.12 (0.54)	0.83	1.17 (0.69)	0.09	0.98 (0.53)	0.07	0.51 (0.74)	0.49
**R**_**eI**_ (cmH_2_O·s/L)	0.02 (0.05)	0.74	0.09 (0.32)	0.78	-0.17 (0.04)	**< 0.01**	-1.49 (0.38)	**< 0.01**	0.19 (0.39)	0.63	1.02 (0.49)	**0.04**	0.71 (0.38)	0.06	0.25 (0.53)	0.64
**ΔR** (cmH_2_O·s/L)	-0.05 (0.03)	0.10	-0.07 (0.22)	0.76	-0.14 (0.03)	**< 0.01**	-0.39 (0.27)	0.15	-0.07 (0.27)	0.80	0.15 (0.35)	0.67	0.27 (0.26)	0.31	0.25 (0.37)	0.49
**X**_**eE**_ (cmH_2_O·s/L)	-0.01 (0.05)	0.75	-0.35 (0.30)	0.24	0.14 (0.04)	**< 0.01**	0.17 (0.37)	0.63	-0.75 (0.36)	**0.04**	-0.58 (0.47)	0.22	-0.09 (0.35)	0.79	0.24 (0.49)	0.63
**X**_**eI**_ (cmH_2_O·s/L)	-0.05 (0.02)	0.06	-0.09 (0.17)	0.58	0.08 (0.02)	**< 0.01**	0.35 (0.20)	0.08	-0.24 (0.20)	0.24	-0.50 (0.25)	**0.05**	-0.31 (0.19)	0.12	-0.01 (0.27)	0.97
**ΔX** (cmH_2_O·s/L)	0.03 (0.03)	0.30	-0.26 (0.21)	0.22	0.06 (0.03)	**0.04**	-0.17 (0.25)	0.50	-0.52 (0.25)	**0.04**	-0.08 (0.33)	0.80	0.21 (0.24)	0.38	0.25 (0.34)	0.48

^**a**^ The term “beta” refers to the regression coefficient estimated by each linear regression model considering an oscillometry parameter (absolute value) as the dependent variable and the individual child characteristics as the independent variable

Bold: *p* value ≤ 0.05

### Associations between individual child characteristics and intra-breath oscillometry parameters at the 3-year visit

Regarding intra-breath oscillometry indices, sex and age had no significant effect on any of the intra-breath indices (Table 4 and Supplementary Table S4). All intra-breath indices showed a linear trend with height and weight, with lower resistance (R) and higher reactance (X) indices as height and weight increased. Higher birth weight was associated with lower R_eE_ and R_eI_. Children exposed to tobacco smoke had lower X_eE_ and ΔX values. The duration of breastfeeding was unexpectedly associated with a higher R_eI_ and a lower X_eI_. Higher parental educational level was associated with lower resistance (R_eE_, R_eI_, ΔR) and higher reactance (X_eE_, X_eI_, ΔX) indices. No statistically significant differences were observed for gestational duration, parental history of asthma, or child allergic sensitization status.

### Associations between history of respiratory illnesses, hospitalisation for respiratory problem and standard and intra-breath oscillometry parameters at the 3-year visit

**Fig. 2:**
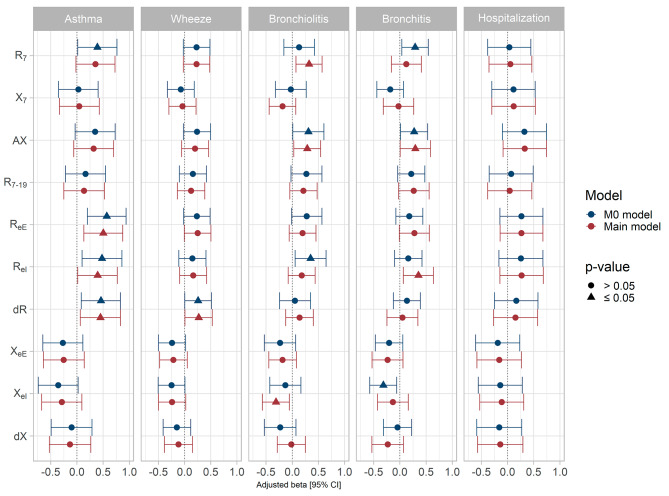
Adjusted associations between each child history of respiratory disease in the first three years of life and each oscillometry parameter measured at 3 years. The M0 models were adjusted on age (continuous), sex and height (continuous) (*n* = 251). The main models were further adjusted on birth weight (continuous), tobacco smoke exposure (assessed by questionnaire at 12 months and 36 months; ever vs. never), breastfeeding at 2 months (binary), parental history of asthma (binary), child allergic sensitization (≥ 1 positive skin prick test vs. none, binary) (*n* = 251). Each oscillometry parameter was re-scaled (divided by its standard deviation) to allow comparisons of the effect estimates across the different oscillometry parameters

The unadjusted analysis between each respiratory outcome and each standard and intra-breath oscillometry parameter are presented in supplementary Table S5. The analyses adjusted on age, sex and height showed a general trend towards an increased R_7_, AX and R_7 − 19_ with each respiratory outcome and statistically significant associations were reached for R_7_ with asthma and bronchiolitis and for AX with bronchiolitis and bronchitis (Table 5; Fig. 2 with re-scaled parameters to allow comparison of the beta estimates). Regarding the intra-breath indices, the M0 model showed a general pattern of association for increased resistance and decreased reactance with each respiratory outcome, and statistically significant associations were observed for R_eI_, R_eE_ and ΔR with asthma, ΔR with wheezing, R_eI_ with bronchitis and X_eI_ with bronchiolitis.

**Table 5:** Age-, sex- and height-adjusted associations (M0 models) between each history of respiratory diseases and each oscillometry parameter measured at 3 years

	Asthma diagnosis ever		Wheezing ever		Bronchiolitis first 2 years		Bronchitis ever		Hospitalisation for respiratory problem	
	Beta (95% CI)^a^	*P* value	Beta (95% CI) ^a^	*P* value	Beta (95% CI) ^a^	*P* value	Beta (95% CI) ^a^	*P* value	Beta (95% CI) ^a^	*P* value
**R**_**7**_ (cmH_2_O·s/L)	0.85 (0.04; 1.66)	**0.04**	0.50 (-0.06; 1.05)	0.08	0.63 (0.08; 1.18)	**0.03**	0.29 (-0.35; 0.92)	0.38	0.07 (-0.83; 0.97)	0.88
**X**_**7**_ (cmH_2_O·s/L)	0.03 (-0.41; 0.47)	0.89	-0.08 (-0.38; 0.21)	0.58	-0.22 (-0.51; 0.08)	0.15	-0.03 (-0.37; 0.31)	0.85	0.13 (-0.35; 0.61)	0.59
**AX** (cmH_2_O/L)	11.72 (-1.25; 24.68)	0.08	8.06 (-0.73; 16.86)	0.07	9.15 (0.33; 17.97)	**0.04**	10.42 (0.37; 20.47)	**0.04**	10.90 (-3.35; 25.15)	0.13
**R**_**7 − 19**_ (cmH_2_O·s/L)	0.15 (-0.21; 0.51)	0.40	0.15 (-0.09; 0.39)	0.23	0.20 (-0.04; 0.45)	0.10	0.25 (-0.02; 0.53)	0.07	0.06 (-0.33; 0.46)	0.75
**R**_**eE**_ (cmH_2_O·s/L)	2.03 (0.71; 3.35)	**< 0.01**	0.84 (-0.07; 1.74)	0.07	0.64 (-0.28; 1.56)	0.17	0.99 (-0.0; 2.03)	0.06	0.95 (-0.52; 2.42)	0.21
**R**_**eI**_ (cmH_2_O·s/L)	1.22 (0.25; 2.19)	**0.01**	0.38 (-0.28; 1.04)	0.26	0.41 (-0.26; 1.08)	0.23	0.90 (0.14; 1.65)	**0.02**	0.65 (-0.42; 1.73)	0.23
**ΔR** (cmH_2_O·s/L)	0.81 (0.15; 1.47)	**0.02**	0.46 (0.00; 0.91)	**0.05**	0.24 (-0.22; 0.69)	0.31	0.09 (-0.43; 0.61)	0.72	0.29 (-0.44; 1.03)	0.43
**X**_**eE**_ (cmH_2_O·s/L)	-0.64 (-1.56; 0.27)	0.17	-0.57 (-1.19; 0.04)	0.07	-0.50 (-1.12; 0.13)	0.12	-0.56 (-1.27; 0.15)	0.12	-0.45 (-1.46; 0.56)	0.38
**X**_**eI**_ (cmH_2_O·s/L)	-0.47 (-0.98; 0.03)	0.06	-0.33 (-0.67; 0.01)	0.06	-0.42 (-0.76; -0.08)	**0.02**	-0.17 (-0.57; 0.22)	0.38	-0.18 (-0.74; 0.37)	0.51
**ΔX** (cmH_2_O·s/L)	-0.17 (-0.82; 0.48)	0.61	-0.25 (-0.69; 0.20)	0.27	-0.08 (-0.52; 0.37)	0.74	-0.39 (-0.89; 0.12)	0.13	-0.26 (-0.98; 0.45)	0.47

^**a**^ The term “beta” refers to the regression coefficient estimated by each linear regression model considering an oscillometry parameter (absolute value) as the dependent variable and asthma (or wheezing or bronchitis or bronchiolitis) as the independent variable

Bold: *p* value ≤ 0.05

The pattern of associations remained in the main model after adjusting for birth weight, tobacco smoke exposure, breastfeeding, parental history of asthma and allergic sensitization (Supplementary Table S6; Fig. 2 with re-scaled parameters). For each respiratory outcome, the strongest magnitude of association was observed for an intra-breath oscillometry parameter: the strongest association for asthma, wheeze, bronchiolitis and bronchitis was observed for R_eI_ (adjusted beta [95% CI] for re-scaled parameter: 0.52 [0.15; 0.90]), ΔR (0.27 [0.01; 0.53]), X_eI_ (-0.30 [-0.56; -0.04]) and R_eI_ (0.35 [0.05; 0.64]), respectively).

Regarding hospitalisation for respiratory problem, none of the association reached the statistical significance, but the patterns of association with both standard (increased AX) and intra-breath (increased R_eE_ and R_eI_) oscillometry parameters are consistent with those observed for history of respiratory illnesses (Supplementary Table S6; Fig. 2 with re-scaled parameters).

## Discussion

We found that a diagnosis of asthma, a history of wheezing or LRTI in the first years of life are associated with subsequent altered lung function measured using standard spectral oscillometry at 3 years, independent of age, height, sex, tobacco smoke exposure, breastfeeding, parental history of asthma or allergic sensitization. The recently introduced intra-breath oscillometric indices showed a trend for stronger associations particularly between asthma diagnosis and wheezing and the volume dependence of resistance (ΔR).

The tidal changes in R may be related to flow-dependance of resistance, and the changes in glottic aperture that narrows during expiration (Supplementary Fig. S1). The eE and eI values and thus their difference in R_10_ (ΔR), are theoretically independent of flow. ΔR reflects the volume-dependence of airway caliber, but is also influenced by intra-breath changes in peripheral mechanical inhomogeneity in the lung. On the other hand, X_10_ becomes slightly more negative at end-inspiration, reflecting a higher apparent respiratory elastance with tidal expansion. The mechanism through which intra-tidal ΔR and ΔX change in children with airway obstruction are likely complex and not well elucidated. However, as shown in Figure S1, in children with increased resistance, ΔR is enhanced. This could be due to volume-dependent changes in airway tone, decreasing during inspiration and returning to a higher baseline level upon expiration [17]. Also, peripheral mechanical inhomogeneity may increase during expiration due to airway narrowing and closure promoted by the decrease in lung volume, possibly leading to expiratory airflow limitation [27]. This inhomogeneity is also suggested by less negative X_10_ values at end-inspiration (Fig. S1B), possibly through recruitment and increased patency of peripheral airways which in contrast to normal lung, reduce the apparent lung elastance on inspiration [20].

A strength of this study is that it assessed both spectral and intra-breath oscillometric measurements of lung function in a population-based epidemiological cohort of preschool children. Previously, the volume-dependence of resistance measured at zero-flow between end-expiration and end-inspiration (ΔR) was found to be a sensitive and specific marker of airway obstruction in preschool children with acute or recurrent wheeze [17]. Intra-breath impedance indices were shown to discriminate between controlled and uncontrolled adult asthmatics, with a better performance than common spectral oscillometry parameters [19]. A potential explanation that has been proposed for the sensitivity of intra-breath indices is that spectral measurements of Z_rs_ which are averaged over the whole breathing cycle, are dependent on breathing pattern and particularly end-expiratory volume, which tends to be highly variable in preschool children [28]. Moreover, the mean resistance values for whole breathing cycles are affected by the changes in glottic aperture during spontaneous breathing. The values of Z_rs_ at zero flow (i.e. at end-inspiration and end-expiration), detected with intra-breath tracking may therefore be less affected by respiratory pattern and changes in upper-airway patency [29].

The acceptability and success rate of lung function measured using oscillometry technique in a population-based cohort of 3-year-old children were good and comparable to previous studies [21], thus confirming that oscillometry is easily performed in pre-school children. In accordance with previous population-based studies [14, 25, 30], height was observed as an important determinants of respiratory impedance. Mean Zscore values for R_7_ and X_7_ observed in our population were 0.5 indicating slightly higher values as compared to the population used as reference in Shackleton et al. [25]. We do not have a clear explanation for that, but the slight offset may be due to differences between populations, differences in measurement devices used and the fact that R_7_-Zscore values were estimated from linear interpolation using R_6_-Zscore and R_8_-Zscore. However, there is no reason to believe that the overall higher outcome measures observed in the SEPAGES population affected the reported associations.

Another strength of this study relies on the deep phenotypic characterization of the SEPAGES cohort, including both spectral and intrabreath oscillometry parameters measured at 3-years for 251 children. In addition, the abundant prospective data in the cohort allowed us to account for a large number of known or potential confounders in the estimated associations. Finally, the study population is rather homogeneous particularly in terms of socio-economic status and ethnicity (97% Caucasian), limiting the risk for confounding bias.

We acknowledge some limitations of the study. Although respiratory symptoms and diseases were repeatedly assessed by a questionnaire administered by an interviewer, the risk for recall bias, difficulty for some parents to differentiate between these respiratory outcomes and diagnostic overlap cannot be totally excluded. However, if significant misclassification errors in symptoms or respiratory conditions had occurred, these would be unrelated to the oscillometry parameters measured at 3 years, and would therefore skew the association results towards the null. Differences in prevalence between the five respiratory health outcomes in our population, from 9.6% for hospitalisation to 38% from bronchitis have a direct impact on statistical power, and mainly explain the lack of statistical significance of the asthma-XeI association even though the magnitude of the association was identical to the bronchitis-XeI statistically significant association as well as the lack of statistical significance of the hospitalisation-AX association even though the magnitude of the association was similar to the bronchitis-AX and bronchiolitis-AX statistically significant associations. This highlights the rationale of our strategy consisting in interpreting the results by looking at the general patterns of association between the different respiratory health outcomes and not limiting the interpretation to the arbitrary 0.05 statistical significance. Finally, although the study was based on strong a priori hypothesis supported by previous studies, the number of associations tested was relatively high (*n* = 40) and we cannot dismiss that part of the associations reported may result from chance findings.

## Conclusions

Our findings indicate a link between a history of early-life wheezing and LRTI and decreased lung function in 3-year-old population-based children as assessed by both spectral and intra-breath oscillometry, the associations of the latter tending to be stronger with early life respiratory illness. Our study supports the relevance of using intra-breath oscillometry parameters as sensitive outcome measures in preschool children in epidemiological cohorts. Given the importance of early-life lung function impairment in subsequent general health, findings from epidemiological research able to characterize early life lung function and its determinants should help inform strategies to prevent chronic respiratory diseases and improve the health of the population.

### Electronic supplementary material

Below is the link to the electronic supplementary material.

Supplementary Material 1

Supplementary Material 2

Supplementary Material 3

Supplementary Material 4

Supplementary Material 5

Supplementary Material 6

Supplementary Material 7

Supplementary Material 8

## Data Availability

The datasets used and analysed during the current study are available from the corresponding author on reasonable request.

## Abbreviations

AX: area of the reactance curve
CV: coefficient of variation
eE: end expiration
eI: end inspiration
FCS: fully conditional specification method
FOT: forced oscillation technique
LRTI: lower respiratory tract illness
P: pressure
R: resistance
R_7_: resistance at 7 Hz
R_10_: resistance at 10 Hz
R_7 − 19_: frequency dependence of the resistance
ReI: end-inspiratory resistance
ReE: end-expiratory resistance
SEPAGES: Suivi de l’Exposition à la Pollution Atmosphérique durant la Grossesse et Effets sur la Santé
V: volume
V’: flow
X: reactance
X_7_: reactance at 7 Hz
X_10_: reactance at 10 Hz
XeI: end-inspiratory reactance
XeE: end-expiratory reactance
Zrs: respiratory impedance
ΔR: change between end-expiratory and end-inspiratory resistance
ΔX: change between end-expiratory and end-inspiratory reactance
