# Supplementary material for: Intra-breath changes in respiratory mechanics are sensitive to history of respiratory illness in preschool children: the SEPAGES cohort
Source: Respir Res. 2024 Feb 24;25:99. doi: 10.1186/s12931-024-02701-9 (PMC10893684; doi:10.1186/s12931-024-02701-9)
Supplement: Supplementary file 1 — Supplementary Material 1 [file 12931_2024_2701_MOESM1_ESM.docx]

**Online supplement**

**Intra-breath changes in respiratory mechanics are sensitive to history of respiratory illness in preschool children:**

**the SEPAGES cohort**

Valérie Siroux ^1^, Anne Boudier^1,2^, Sarah Lyon-Caen^1^, Joane Quentin^1^, Yoann Gioria^1^, Zoltán Hantos^3,4^, Rémy Slama^1^, Isabelle Pin^1,2^, Sam Bayat^5^

**FIGURES LEGEND**

**FIGURE S1_** Examples of the intra-breath data in a child with normal airway resistance and in a child with elevated airway resistance

**FIGURE S2_** Directed Acyclic Graph (DAG)

The green circle represents the “exposure” variables (respiratory diseases/symptoms). The blue circle (with a I) represents the outcome (oscillometry parameters). The red circles represent the Minimal Sufficient Adjustment Set (MSAS).

**FIGURE S3_** Analysis of linear model assumptions with residuals plots assessing linearity of the association (left), homogeneity of the variance of the residuals (centre) and normality of the residuals (right) for each linear regression model using the main models (fully adjusted).

**TABLE S1_** Correlation matrix between oscillometry parameters

|  | **R_7_** (cmH_2_O·s/L) | **X_7_** (cmH_2_O·s/L) | **AX** (cmH_2_O/L) | **R_7-19_** (cmH_2_O·s/L) | **R_eE_** (cmH_2_O·s/L) | **R_eI_** (cmH_2_O·s/L) | **ΔR** (cmH_2_O·s/L) | **X_eE_** (cmH_2_O·s/L) | **X_eI_** (cmH_2_O·s/L) | **ΔX** (cmH_2_O·s/L) |
| --- | --- | --- | --- | --- | --- | --- | --- | --- | --- | --- |
| **R_7_** (cmH_2_O·s/L) | 1 | -0.66  *<0.01* | 0.77  *<0.01* | 0.44  *<0.01* | 0.67  *<0.01* | 0.65  *<0.01* | 0.42  *<0.01* | -0.49  *<0.01* | -0.47  *<0.01* | -0.32  *<0.01* |
| **X_7_** (cmH_2_O·s/L) |  | 1 | -0.73  *<0.01* | -0.67  *<0.01* | -0.49  *<0.01* | -0.47  *<0.01* | -0.31  *<0.01* | 0.61  *<0.01* | 0.58  *<0.01* | 0.42  *<0.01* |
| **AX** (cmH_2_O/L) |  |  | 1 | 0.56  *<0.01* | 0.63  *<0.01* | 0.65  *<0.01* | 0.34  *<0.01* | -0.63  *<0.01* | -0.55  *<0.01* | -0.48  *<0.01* |
| **R_7-19_** (cmH_2_O·s/L) |  |  |  | 1 | 0.29  *<0.01* | 0.31  *<0.01* | 0.14  *<0.01* | -0.53  *<0.01* | -0.45  *<0.01* | -0.41  *<0.01* |
| **R_eE_** (cmH_2_O·s/L) |  |  |  |  | 1 | 0.88  *<0.01* | 0.74  *<0.01* | -0.67  *<0.01* | -0.59  *<0.01* | -0.50  *<0.01* |
| **R_eI_** (cmH_2_O·s/L) |  |  |  |  |  | 1 | 0.34  *<0.01* | -0.62  *<0.01* | -0.63  *<0.01* | -0.39  *<0.01* |
| **ΔR** (cmH_2_O·s/L) |  |  |  |  |  |  | 1 | -0.46  *<0.01* | -0.27  *<0.01* | -0.45  *<0.01* |
| **X_eE_** (cmH_2_O·s/L) |  |  |  |  |  |  |  | 1 | 0.74  *<0.01* | 0.84  *<0.01* |
| **X_eI_** (cmH_2_O·s/L) |  |  |  |  |  |  |  |  | 1 | 0.27  *<0.01* |
| **ΔX** (cmH_2_O·s/L) |  |  |  |  |  |  |  |  |  | 1 |

**TABLE S2_** Associations between the respiratory symptoms and diseases

|  | **Asthma diag** | **Wheeze**  **No Yes** | **Bronchiolitis**  **No Yes** | **Bronchitis**  **No Yes** |
| --- | --- | --- | --- | --- |
| Asthma diag, %  *P value (χ^2^)* | -- | 1.2 33.3  *<0.01* | 5.3 24.2  *<0.01* | 8.6 24.5  *<0.01* |
| Wheeze, %  *P value (χ^2^)* |  | -- | 16.1 63.7  *<0.01* | 27.4 56.6  *<0.01* |
| Bronchiolitis, %  *P value (χ^2^)* |  |  | -- | 35.6 45.3  *0.20* |
| Bronchitis, %  *P value (χ^2^)* |  |  |  | -- |

**TABLE S3_** Unadjusted associations between individual child characteristics and standard oscillometry parameters at the 3-year visit (n=251)

|  |  | **R_7_** | |  | **X_7_** | |  | **AX** |  | **R_7-19_** |
| --- | --- | --- | --- | --- | --- | --- | --- | --- | --- | --- |
|  | **n** | **cmH_2_O·s/L**  **m±sd** | **Z-score, m±sd** |  | **cmH_2_O·s/L**  **m±sd** | **Z-score, m±sd** |  | **cmH_2_O/L**  **m±sd** |  | **cmH_2_O/L**  **m±sd** |
| **Sex,**  Boys  Girls  P | 136  115 | 11.8 ± 2.2  11.9 ± 2.2  0.72 | 0.55 ± 0.92  0.54 ± 0.87  0.90 |  | -4.2 ± 1.2  -4.0 ± 1.1  0.32 | 0.64 ± 0.70  0.48 ± 0.65  0.07 |  | 75.1 ± 30.7  71.1 ± 37.4  0.36 |  | 1.38 ± 0.92  1.04 ± 0.90  **<0.01** |
| **Age**  <36.5  [36.5-37.8[  ≥37.8  P | 86  86  79 | 11.9 ± 2.3  11.8 ± 2.2  11.7 ± 2.0  0.75 | 0.56 ±0.95 0.55 ± 0.88  0.52 ± 0.85 0.94 |  | -4.05 ± 1.23  -4.15 ± 1.16  -4.16 ± 1.10 0.78 | 0.49 ± 0.72  0.58 ± 0.68 0.63 ± 0.65  0.40 |  | 74.4 ± 36.4  74.2 ± 34.0  71.0 ± 31.1  0.77 |  | 1.16 ± 0.88  1.25 ± 0.94  1.27 ± 0.96  0.72 |
| **Height**  <94 cm  94-96.4 cm  ≥96.5 cm  P | 74  80  97 | 12.5 ± 2.0  12.0 ± 2.2  11.1 ± 2.1 **<0.01** | 0.64 ± 0.80  0.59 ± 0.92  0.43 ± 0.94  0.26 |  | -4.38 ± 1.07  -4.34 ± 1.27  -3.73 ± 1.03  **<0.01** | 0.52 ± 0.60  0.67 ± 0.75  0.51 ± 0.68  0.26 |  | 78.8 ± 36.7  77.4 ±34.3  65.6 ± 30.1  **0.02** |  | 1.20 ± 0.86  1.36 ± 1.05  1.14 ± 0.86  0.29 |
| **Weight**  <14 kg  14 to 15.2 kg  ≥15.3 kg  P | 80  78  93 | 12.1 ± 2.1  11.7 ± 1.8  11.6 ± 2.5  0.27 | 0.56 ± 0.85  0.50 ± 0.77  0.57 ± 1.03  0.84 |  | -4.20 ± 1.00  -4.15 ± 1.15  -4.01 ± 1.29  0.53 | 0.50 ± 0.60  0.56 ± 0.68  0.62 ± 0.74  0.53 |  | 78.2 ± 37.5  68.5 ± 29.4  73.0 ± 34.0  0.20 |  | 1.21 ± 0.86  1.20 ± 1.01  1.27 ± 0.91  0.87 |
| **Parental educational level**  < graduate level  graduate  p | 66  185 | 12.1 ± 2.0  11.7 ± 2.2  0.17 | 0.68 ± 0.79  0.49 ± 0.92  0.14 |  | -4.22 ± 1.13  -4.08 ± 1.17  0.42 | 0.63 ± 0.66  0.54 ± 0.69  0.38 |  | 79.7 ± 33.4  71.0 ± 33.9  0.07 |  | 1.30 ± 0.88  1.20 ± 0.94  0.49 |
| **Gestational duration**  >37 weeks of amenorrhea  ≤37 weeks of amenorrhea  p | 231  20 | 11.8 ± 2.1  11.6 ± 2.6  0.59 | 0.55 ± 0.90  0.43 ± 0.89  0.54 |  | -4.14 ± 1.15  -3.90 ± 1.29  0.38 | 0.58 ± 0.68  0.39 ±0.71  0.24 |  | 73.3 ± 32.6  73.1 ± 47.2  0.98 |  | 1.22 ± 0.92  1.26 ± 0.96  0.87 |
| **Birth weight**  <3160 g  3160 to 3480 g  ≥3480 g  p | 80  86  85 | 12.2 ± 2.1  11.9 ± 2.2  11.3 ± 2.2  **0.02** | 0.66 ±0.77  0.57 ± 0.94  0.40 ± 0.95  0.16 |  | -4.11 ± 1.10  -4.24 ± 1.27  -4.00 ± 1.09  0.41 | 0.48 ± 0.65  0.62 ± 0.74  0.58 ± 0.65  0.42 |  | 74.7 ± 36.2  76.0 ± 34.7  69.2 ±30.7  0.38 |  | 1.12 ± 0.80  1.26 ± 0.99  1.29 ± 0.96  0.47 |
| **Still breastfeeding at 2 months**  No  Yes  p | 29  221 | 11.2 ± 2.0  11.9 ± 2.2  0.12 | 0.33 ± 0.86  0.57 ± 0.89  0.18 |  | -3.64 ± 0.74  -4.17 ± 1.18  **0.02** | 0.32 ± 0.54  0.59 ± 0.69  **0.05** |  | 64.1 ± 27.4  74.1 ±34.2  0.13 |  | 0.95 ± 0.77  1.26 ± 0.93  0.10 |
| **Tobacco smoke exposure any time since birth**  No  Yes  p | 193  56 | 11.7 ± 2.1  12.1 ± 2.6  0.21 | 0.51 ± 0.87  0.63 ± 0.99  0.38 |  | -4.03 ± 1.08  -4.39 ± 1.35  **0.04** | 0.52 ± 0.65  0.69 ± 0.76  0.09 |  | 70.5 ± 30.5  82.6 ± 42.9  **0.02** |  | 1.19 ± 0.91  1.37 ± 0.99  0.18 |
| **Parental history of asthma**  No  Yes  p | 166  64 | 11.7 ± 2.3  12.1 ± 1.9  0.22 | 0.48 ± 0.95  0.69 ± 0.77  0.13 |  | -4.12 ± 1.22  -4.15 ± 1.24  0.86 | 0.55 ± 0.72  0.59 ± 0.65  0.72 |  | 71.5 ± 35.0  78.0 ±32.7  0.20 |  | 1.17 ± 0.95  1.30 ± 0.96  0.35 |
| **Positive skin prick test**  No  Yes, one or more  p | 203  27 | 11.7 ± 2.2  12.5 ± 1.9  0.06 | 0.48 ± 0.91  0.85 ± 0.72  **0.04** |  | -4.07 ± 1.16  -4.28 ±1.19  0.37 | 0.53 ±0.68  0.66 ± 0.67  0.36 |  | 71.7 ±34.7  85.8 ± 30.3  **0.05** |  | 1.17 ± 0.88  1.37 ± 1.05  0.30 |

Bold: p value ≤0.05

**TABLE S4_** Associations between individual child characteristics and intra-breath oscillometry parameters measured at the 3-year visit (n=251)

|  |  | **R_eE_ (cmH_2_O·s/L)** | **R_eI_ (cmH_2_O·s/L)** | **ΔR (cmH_2_O·s/L)** | **X_eE_ (cmH_2_O·s/L)** | **X_eI_ (cmH_2_O·s/L)** | **ΔX (cmH_2_O·s/L)** |
| --- | --- | --- | --- | --- | --- | --- | --- |
|  | **n** | **m±sd** | **m±sd** | **m±sd** | **m±sd** | **m±sd** | **m±sd** |
| **Sex,**  Boys  Girls  p | 136  115 | 13.4 ± 3.3  13.4 ± 3.8  0.96 | 10.6 ± 2.4  10.5 ± 2.8  0.78 | 2.86 ± 1.65  2.92 ± 1.91  0.76 | -3.62 ± 2.33  -3.26 ± 2.44  0.24 | -3.01 ± 1.23  -2.92 ± 1.42  0.58 | -0.61 ± 1.64  -0.34 ± 1.69  0.22 |
| **Age**  <36.5  [36.5-37.8[  ≥37.8  p | 86  86  79 | 14.0 ± 4.12  13.3 ± 3.69  13.0 ± 2.68  0.18 | 10.68 ± 2.98  10.59 ± 2.63  10.32 ± 1.94  0.64 | 3.29 ± 1.92  2.71 ± 1.78  2.64 ± 1.52  **0.03** | -3.53 ± 2.75  -3.39 ± 2.38  -3.44 ± 1.96  0.92 | -2.98 ± 1.28  -2.85 ± 1.38  -3.10 ± 1.30  0.48 | -0.56 ± 1.90  -0.54 ± 1.78  -0.35 ± 1.22  0.68 |
| **Height**  <94 cm  94-96.4 cm  ≥96.5 cm  p | 74  80  97 | 14.6 ± 3.86  13.6 ± 3.74  12.3 ± 2.87  **<0.01** | 11.2 ± 2.87  10.6 ± 2.64  10.0 ± 2.12  **0.01** | 3.42 ± 1.83  3.03 ± 1.75  2.36 ± 1.60  **<0.01** | -3.84 ± 2.63  -3.79 ± 2.59  -2.90 ± 1.89  **0.01** | -3.31 ± 1.46  -3.10 ± 1.34  -2.60 ± 1.10  **<0.01** | -0.52 ± 1.93  -0.68 ± 1.76  -0.30 ± 1.33  0.31 |
| **Weight**  <14 kg  14 to 15.2 kg  ≥15.3 kg  p | 80  78  93 | 14.7 ± 4.11  12.9 ± 2.71  12.8 ± 3.49  **<0.01** | 11.4 ± 2.95  10.1 ± 2.05  10.2 ± 2.43  **<0.01** | 3.27 ± 1.85  2.84 ± 1.53  2.59 ± 1.85  **0.04** | -3.73 ± 2.43  -3.14 ± 1.94  -3.50 ± 2.67  0.30 | -3.31 ± 1.38  -2.71 ± 1.16  -2.89 ± 1.35  **0.01** | -0.41 ± 1.82  -0.43 ± 1.44  -0.60 ± 1.72  0.71 |
| **Parental educational level**  < graduate level  graduate  p | 66  185 | 14.5 ± 3.73  13.0 ± 3.45  **<0.01** | 11.2 ± 2.55  10.3 ± 2.53  **0.01** | 3.29 ± 1.88  2.74 ± 1.71  **0.03** | -4.22 ± 2.73  -3.18 ± 2.20  **<0.01** | -3.20 ± 1.40  -2.89 ± 1.28  0.10 | -1.02 ± 1.96  -0.29 ± 1.51  **<0.01** |
| **Gestational duration**  >37 weeks of amenorrhea  ≤37 weeks of amenorrhea  p | 231  20 | 13.4 ± 3.45  13.76 ± 4.89  0.66 | 10.50 ± 2.47  10.91 ± 3.50  0.49 | 2.89 ± 1.75  2.85 ± 2.05  0.92 | -3.46 ± 2.31  -3.48 ± 3.17  0.97 | -2.98 ± 1.31  -2.89 ±1.50  0.77 | -0.48 ± 1.63  -0.59 ± 2.05  0.77 |
| **Birth weight**  <3160 g  3160 to 3480 g  ≥3480 g  p | 80  86  85 | 13.9 ± 3.6  13.7 ± 3.8  12.7 ± 3.3  0.07 | 10.9 ± 2.6  10.7 ±2.8  10.0 ± 2.2  0.06 | 2.98 ± 1.82  2.98 ± 1.86  2.70 ± 1.63  0.50 | -3.30 ± 2.26  -3.51 ± 2.37  -3.55 ± 2.53  0.77 | -2.99 ± 1.27  -3.11 ± 1.52  -2.80 ±1.12  0.31 | -0.31 ± 1.66  -0.40 ± 1.62  -0.74 ± 1.71  0.21 |
| **Still breastfeeding at 2 months**  No  Yes  p | 29  221 | 12.3 ± 2.51  13.5 ±3.62  0.09 | 9.60 ±1.83  10.6 ±2.56  **0.04** | 2.75 ± 1.46  2.90 ± 1.81  0.67 | -2.93 ± 1.68  -3.51 ± 2.46  0.22 | -2.51 ± 0.99  -3.01 ± 1.30  **0.05** | -0.42 ± 1.13  -0.51 ± 1.72  0.80 |
| **Tobacco smoke exposure any time since birth**  No  Yes  p | 193  56 | 13.4 ± 3.35  13.5 ± 4.34  0.83 | 10.5 ± 2.40  10.7 ± 3.10  0.63 | 2.91 ± 1.76  2.84 ±1.84  0.80 | -3.30 ± 2.14  -4.05 ± 3.06  **0.04** | -2.92 ± 1.29  -3.15 ± 1.42  0.24 | -0.38 ± 1.54  -0.90 ± 2.01  **0.04** |
| **Parental history of asthma**  No  Yes  p | 166  64 | 13.2 ± 3.61  14.2 ± 3.57  0.07 | 10.3 ± 2.55  11.0 ± 2.62  0.06 | 2.85 ± 1.83  3.12 ±1.66  0.31 | -3.40 ± 2.43  -3.50 ± 2.24  0.79 | -2.88 ± 1.28  -3.18 ± 1.44  0.12 | -0.53 ± 1.68  -0.31 ± 1.62  0.38 |
| **Positive skin prick test**  No  Yes, one or more  p | 203  27 | 13.3 ± 3.64  13.8 ± 3.41  0.49 | 10.5 ± 2.62  10.7 ± 2.43  0.64 | 2.86 ± 1.78  3.12 ± 1.80  0.49 | -3.49 ± 2.40  -3.25 ± 2.48  0.63 | -2.96 ± 1.31  -2.97 ± 1.55  0.97 | -0.53 ± 1.68  -0.28 ± 1.75  0.48 |

Bold: p value ≤0.05

**TABLE S5_** Unadjusted-associations between each history of respiratory diseases and oscillometry parameters measured at 3 years

Bold : P-value<0.05

|  | **Asthma diagnosis ever** | | | **Wheezing ever** | | | **Bronchiolitis first 2 years** | | | **Bronchitis ever** | | | **Hospitalisation for respiratory problem** | | | |
| --- | --- | --- | --- | --- | --- | --- | --- | --- | --- | --- | --- | --- | --- | --- | --- | --- |
|  | **No**  **(n=221)** | **Yes**  **(n=30)** | **P value** | **No**  **(n=166)** | **Yes**  **(n=84)** | **P value** | **No**  **(n=150)** | **Yes**  **(n=91)** | **P value** | **No**  **(n=198)** | **Yes**  **(n=53)** | **P**  **value** | **No**  **(n=227)** | **Yes**  **(n=24)** | **P**  **value** |  |
| **R_7_** (cmH_2_O·s/L), mean(sd) | 11.73 (2.23) | 12.37 (1.70) | 0.13 | 11.63 (2.18) | 12.16 (2.16) | 0.07 | 11.62 (2.24) | 12.12 (2.08) | 0.09 | 11.76 (2.20) | 11.98 (2.10) | 0.50 | 11.81 (2.23) | 11.81 (1.69) | 0.99 |  |
| **X_7_** (cmH_2_O·s/L), mean(sd) | -4.12 (1.19) | -4.09 (0.84) | 0.91 | -4.06 (1.15) | -4.20 (1.16) | 0.36 | -4.03 (1.11) | -4.24 (1.22) | 0.17 | -4.11 (1.16) | -4.13 (1.16) | 0.89 | -4.12 (1.19) | -4.05 (0.83) | 0.76 |  |
| **AX** (cmH_2_O/L), mean(sd) | 71.92 (34.4) | 83.27 (28.2) | 0.08 | 70.0 (33.0) | 79.38 (35.0) | **0.04** | 70.11 (33.4) | 78.95 (34.7) | **0.05** | 71.13 (33.5) | 81.27 (34.3) | **0.05** | 72.16 (34.18) | 83.85 (29.89) | 0.11 |  |
| **R_7-19_** (cmH_2_O·s/L), mean(sd) | 1.19 (0.93) | 1.43 (0.79) | 0.19 | 1.15 (0.88) | 1.35 (0.98) | 0.11 | 1.14 (0.86) | 1.39 (1.00) | **0.04** | 1.17 (0.90) | 1.43 (0.99) | 0.07 | 1.21 (0.93) | 1.34 (0.91) | 0.51 |  |
| **R_eE_** (cmH_2_O·s/L), mean(sd) | 13.23 (3.55) | 14.83 (3.52) | **0.02** | 13.14 (3.66) | 14.00 (3.35) | 0.07 | 13.29 (3.80) | 13.70 (3.30) | 0.39 | 13.24 (3.55) | 14.08 (3.63) | 0.13 | 13.36 (3.62) | 14.04 (3.13) | 0.37 |  |
| **R_eI_** (cmH_2_O·s/L), mean(sd) | 10.41 (2.56) | 11.46 (2.36) | **0.03** | 10.39 (2.66) | 10.83 (2.34) | 0.20 | 10.44 (2.68) | 10.75 (2.40) | 0.37 | 10.36 (2.54) | 11.19 (2.54) | **0.03** | 10.48 (2.61) | 11.07 (1.97) | 0.28 |  |
| **ΔR** (cmH_2_O·s/L), mean(sd) | 2.82 (1.76) | 3.37 (1.80) | 0.11 | 2.75 (1.75) | 3.17 (1.77) | 0.07 | 2.85 (1.81) | 2.95 (1.71) | 0.67 | 2.88 (1.69) | 2.89 (2.03) | 0.99 | 2.88 (1.77) | 2.97 (1.82) | 0.81 |  |
| **X_eE_** (cmH_2_O·s/L), mean(sd) | -3.38 (2.42) | -3.98 (2.08) | 0.20 | -3.24 (2.42) | -3.89 (2.27) | **0.04** | -3.31 (2.51) | -3.77 (2.21) | 0.15 | -3.34 (2.30) | -3.86 (2.64) | 0.16 | -3.41 (2.44) | -3.87 (1.73) | 0.38 |  |
| **X_eI_** (cmH_2_O·s/L), mean(sd) | -2.91 (1.30) | -3.38 (1.36) | 0.07 | -2.84 (1.29) | -3.23 (1.33) | **0.03** | -2.81 (1.29) | -3.23 (1.35) | **0.02** | -2.93 (1.31) | -3.11 (1.36) | 0.39 | -2.95 (1.36) | -3.20 (0.83) | 0.37 |  |
| **ΔX** (cmH_2_O·s/L), mean(sd) | -0.47 (1.70) | -0.60 (1.33) | 0.69 | -0.40 (1.71) | -0.65 (1.57) | 0.25 | -0.49 (1.72) | -0.54 (1.60) | 0.81 | -0.41 (1.61) | -0.75 (1.83) | 0.19 | -0.47 (1.70) | -0.66 (1.32) | 0.58 |  |

**TABLE S6_** Fully-adjusted^a^ associations (main models) between each history of respiratory diseases and oscillometry parameters measured at 3 years

|  | **Asthma diagnosis ever** | | **Wheezing ever** | | | **Bronchiolitis first 2 years** | | **Bronchitis ever** | | **Hospitalisation for respiratory problem** | |
| --- | --- | --- | --- | --- | --- | --- | --- | --- | --- | --- | --- |
|  | **Beta**  **(95% CI)^b^** | **P value** | **Beta**  **(95% CI)^b^** | **P**  **value** | **Beta**  **(95% CI) ^b^** | | **P value** | **Beta**  **(95% CI) ^b^** | **P**  **value** | **Beta**  **(95% CI) ^b^** | **P**  **value** |
| **R_7_** (cmH_2_O·s/L) | 0.77  (-0.04 ; 1.58) | 0.06 | 0.50  (-0.05 ; 1.05) | 0.08 | 0.70  (0.15 ; 1.24) | | **0.01** | 0.27  (-0.36 ; 0.89) | 0.40 | 0.12  (-0.78 ; 1.01) | 0.80 |
| **X_7_** (cmH_2_O·s/L) | 0.05  (-0.39 ; 0.49) | 0.82 | -0.05  (-0.35 ; 0.25) | 0.75 | -0.22  (-0.51 ; 0.08) | | 0.15 | -0.03  (-0.37 ; 0.30) | 0.85 | 0.13  (-0.35 ; 0.61) | 0.59 |
| **AX** (cmH_2_O/L) | 10.85  (-2.06 ; 23.75) | 0.10 | 6.79  (-1.95 ; 15.54) | 0.13 | 9.66  (0.98 ; 18.36) | | **0.03** | 9.96  (0.14 ; 19.78) | **0.05** | 11.13  (-2.95 ; 25.20) | 0.12 |
| **R_7-19_** (cmH_2_O·s/L) | 0.13  (-0.24 ; 0.49) | 0.49 | 0.11  (-0.13 ; 0.36) | 0.36 | 0.20  (-0.04 ; 0.44) | | 0.11 | 0.25  (-0.03 ; 0.52) | 0.08 | 0.04  (-0.36 ; 0.44) | 0.85 |
| **R_eE_** (cmH_2_O·s/L) | 1.80  (0.47 ; 3.13) | **<0.01** | 0.89  (-0.02 ; 1.80) | 0.05 | 0.71  (-0.21 ; 1.62) | | 0.13 | 0.99  (-0.03 ; 2.01) | 0.06 | 0.95  (-0.52 ; 2.42) | 0.20 |
| **R_eI_** (cmH_2_O·s/L) | 1.01  (0.04 ; 1.97) | **0.04** | 0.42  (-0.24 ; 1.08) | 0.21 | 0.46  (-0.20 ; 1.12) | | 0.17 | 0.90  (0.17 ; 1.64) | **0.02** | 0.68  (-0.38 ; 1.74) | 0.21 |
| **ΔR** (cmH_2_O·s/L) | 0.79  (0.11 ; 1.47) | **0.02** | 0.47  (0.01 ; 0.93) | **0.04** | 0.24  (0.71 ; 0.30) | | 0.30 | 0.08  (-0.44 ; 0.61) | 0.75 | 0.27  (-0.48 ; 1.01) | 0.48 |
| **X_eE_** (cmH_2_O·s/L) | -0.60  (-1.53 ; 0.33) | 0.20 | -0.51  (-1.14 ; 0.12) | 0.11 | -0.44  (-1.07 ; 0.19) | | 0.17 | -0.56  (-1.27 ; 0.14) | 0.12 | -0.38  (-1.39 ; 0.63) | 0.46 |
| **X_eI_** (cmH_2_O·s/L) | -0.38  (-0.89 ; 0.12) | 0.14 | -0.31  (-0.66 ; 0.03) | 0.07 | -0.41  (-0.75 ; -0.07) | | **0.02** | -0.18  (-0.56 ; 0.21) | 0.37 | -0.15  (-0.70 ; 0.41) | 0.60 |
| **ΔX** (cmH_2_O·s/L) | -0.22  (-0.88 ; 0.44) | 0.51 | -0.20  (-0.64 ; 0.25) | 0.39 | -0.03  (-0.48 ; 0.42) | | 0.90 | -0.39  (-0.89 ; 0.11) | 0.13 | -0.23  (-0.95 ; 0.49) | 0.52 |

^a^Adjusted on age (continuous), sex, height (continuous), tobacco smoke exposure (ever vs. never), breastfeeding (still breastfeeding at 2 months, yes/no), parental history of asthma (binary), child allergic sensitization (≥1 positive skin prick test vs. none) and birth weight (continuous);

^b^ The term “beta” refers to the regression coefficient estimated by each linear regression model considering an oscillometry parameter as the dependent variable and asthma (or wheezing or bronchitis or bronchiolitis) as the independent variable.

Bold: P-value<0.05
