# Supplementary material for: Intra-breath changes in respiratory mechanics are sensitive to history of respiratory illness in preschool children: the SEPAGES cohort
Source: Respir Res. 2024 Feb 24;25:99. doi: 10.1186/s12931-024-02701-9 (PMC10893684; doi:10.1186/s12931-024-02701-9)
Supplement: Supplementary file 2 — Supplementary Material 2 [file 12931_2024_2701_MOESM2_ESM.docx]

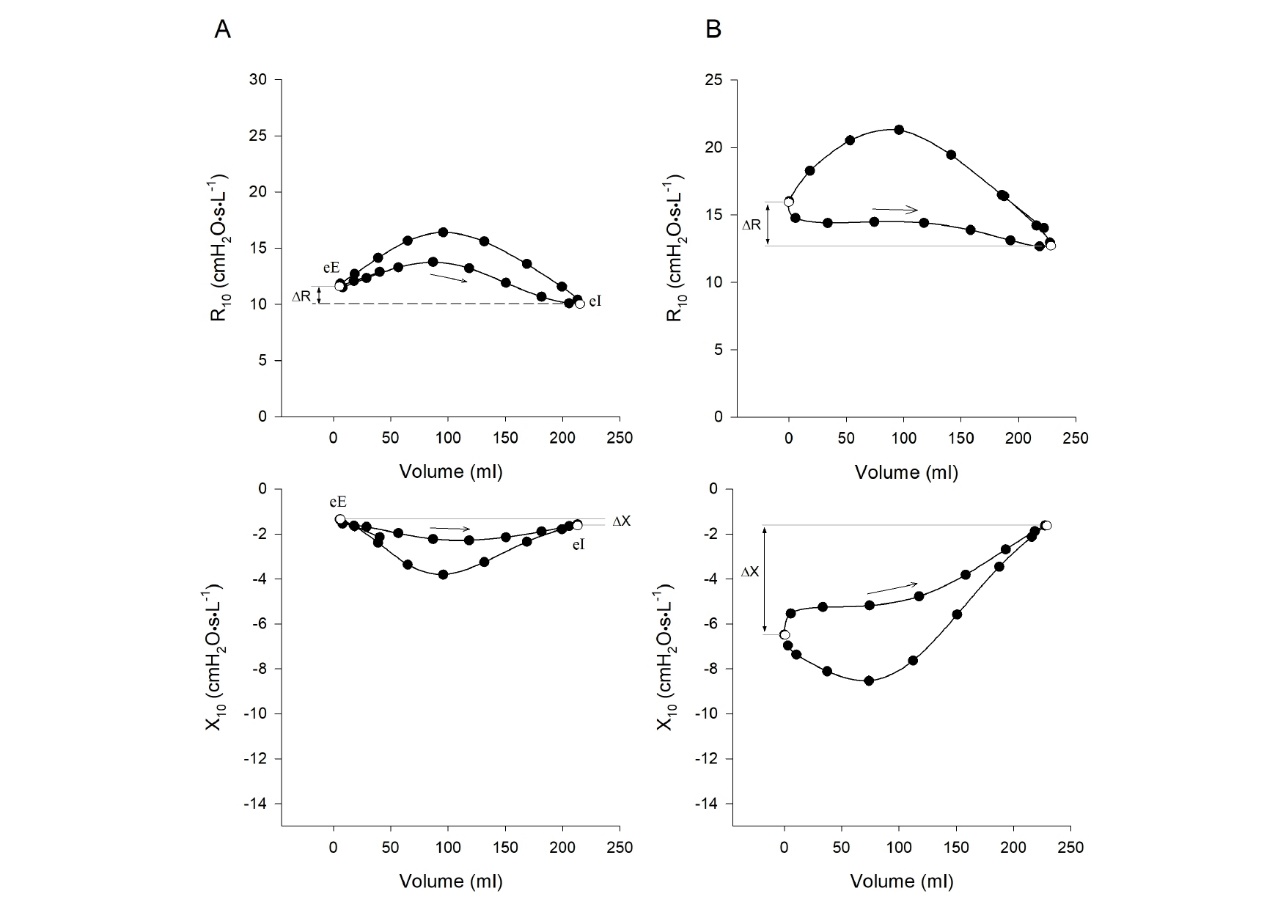


**Additional File 2**: “5_Fig S1_example_intrabreath_data”

FIGURE S1_ Examples of the intra-breath data in a child with normal airway resistance and in a child with elevated airway resistance
