# Supplementary material for: Intra-breath changes in respiratory mechanics are sensitive to history of respiratory illness in preschool children: the SEPAGES cohort
Source: Respir Res. 2024 Feb 24;25:99. doi: 10.1186/s12931-024-02701-9 (PMC10893684; doi:10.1186/s12931-024-02701-9)
Supplement: Supplementary file 3 — Supplementary Material 3 [file 12931_2024_2701_MOESM3_ESM.docx]

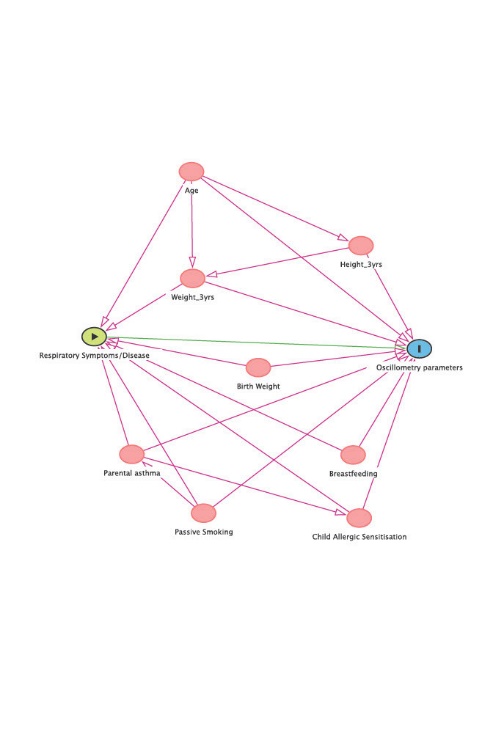


**Additional File 3:** “6_Fig S2_DAG”

FIGURE S2_ Directed Acyclic Graph (DAG)

The green circle represents the “exposure” variables (respiratory diseases/symptoms). The blue circle (with a I) represents the outcome (oscillometry parameters). The red circles represent the Minimal Sufficient Adjustment Set (MSAS).
