# Supplementary material for: Intra-breath changes in respiratory mechanics are sensitive to history of respiratory illness in preschool children: the SEPAGES cohort
Source: Respir Res. 2024 Feb 24;25:99. doi: 10.1186/s12931-024-02701-9 (PMC10893684; doi:10.1186/s12931-024-02701-9)
Supplement: Supplementary file 5 — Supplementary Material 5 [file 12931_2024_2701_MOESM5_ESM.docx]

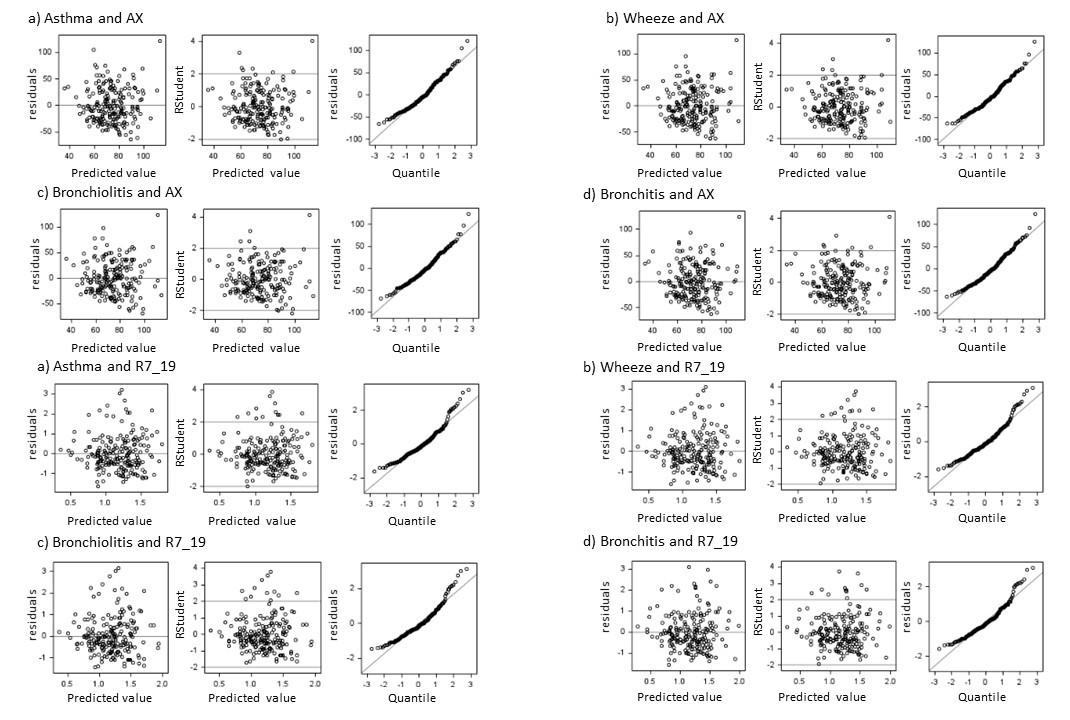


**Additional Figure 5 :** “7b_Fig S3_Test linear models _AX - R7-19”

FIGURE S3b_ Analysis of linear model assumptions with residuals plots assessing linearity of the association (left), homogeneity of the variance of the residuals (centre) and normality of the residuals (right) for each linear regression model using the main models (fully adjusted).

Results for AX and R7-19
